# A Seriation Approach for Visualization-Driven Discovery of Co-Expression Patterns in Serial Analysis of Gene Expression (SAGE) Data

**DOI:** 10.1371/journal.pone.0003205

**Published:** 2008-09-12

**Authors:** Olena Morozova, Vyacheslav Morozov, Brad G. Hoffman, Cheryl D. Helgason, Marco A. Marra

**Affiliations:** 1 Genome Sciences Centre, BC Cancer Agency, Vancouver, British Columbia, Canada; 2 Donnelly Centre for Cellular and Biomolecular Research, University of Toronto, Toronto, Ontario, Canada; 3 Department of Cancer Endocrinology, BC Cancer Research Centre, Vancouver, British Columbia, Canada; Harvard School of Public Health, United States of America

## Abstract

**Background:**

Serial Analysis of Gene Expression (SAGE) is a DNA sequencing-based method for large-scale gene expression profiling that provides an alternative to microarray analysis. Most analyses of SAGE data aimed at identifying co-expressed genes have been accomplished using various versions of clustering approaches that often result in a number of false positives.

**Principal Findings:**

Here we explore the use of seriation, a statistical approach for ordering sets of objects based on their similarity, for large-scale expression pattern discovery in SAGE data. For this specific task we implement a seriation heuristic we term ‘progressive construction of contigs’ that constructs local chains of related elements by sequentially rearranging margins of the correlation matrix. We apply the heuristic to the analysis of simulated and experimental SAGE data and compare our results to those obtained with a clustering algorithm developed specifically for SAGE data. We show using simulations that the performance of seriation compares favorably to that of the clustering algorithm on noisy SAGE data.

**Conclusions:**

We explore the use of a seriation approach for visualization-based pattern discovery in SAGE data. Using both simulations and experimental data, we demonstrate that seriation is able to identify groups of co-expressed genes more accurately than a clustering algorithm developed specifically for SAGE data. Our results suggest that seriation is a useful method for the analysis of gene expression data whose applicability should be further pursued.

## Introduction

With the advent of high throughput technologies, large-scale gene expression studies have become routine in many biological laboratories. Two conceptually different approaches to high throughput gene expression profiling are microarrays [1] and tag sequencing-based methods, such as Serial Analysis of Gene Expression (SAGE) [2]. While both of these gene expression platforms can generate large genome-wide expression data sets, making full use of the data is still an important bioinformatic challenge [3]. A common aim of high throughput gene expression studies is to identify genes with similar expression profiles since such genes may be functionally related and thus may be used to predict functions of unknown genes. This aim has been most often addressed by various versions of clustering analysis that group genes into clusters with correlations among their expression values [4], [5]. Currently available clustering methods show variable success at identifying functionally-relevant gene groupings [6]–[8].

While microarray studies assess gene expression levels by measuring hybridization intensities to the relevant probes [1], SAGE studies use portions of cDNA transcripts known as SAGE tags that are concatenated, cloned, and sequenced to provide a quantitative measure of the transcripts levels in the cell [2]. The use of SAGE had been until recently limited by the sequencing cost and laborious steps inherent in the cloning procedure. However, with modern advances in sequencing technologies, SAGE-related methods have become more cost-effective and are gaining popularity owing to some technological advantages they offer over microarrays [9]. In particular, SAGE does not rely on previous knowledge of gene structure. In addition, it has been suggested that SAGE studies are more robust, and require fewer replicates than microarray studies [9], [10]. Generally, SAGE data have been subjected to the same clustering methods as microarray data [11]. However, more appropriate distance measures accounting for the discreet, Poisson-distributed structure of SAGE data have been shown to produce better clustering results than those achieved with conventional Euclidian or Pearson similarity measures routinely used in microarray data clustering [12]. A successful clustering method for SAGE termed PoissonC accounts for the categorical structure of SAGE data by using the Chi-square statistic and the Poisson distribution in a K-means clustering procedure [12].

A notorious feature of gene expression datasets affecting the performance of cluster analysis is high data dimensionality whereby the expression of many genes is assayed over a small number of experimental conditions (time points). This leads to failure of common statistical methods to distinguish real correlation patterns from spurious ones [3], [5], and necessitates the development of alternative approaches for identifying co-expressed genes. Here we explore whether a reordering rather than grouping approach can be used for the identification of co-expressed genes in gene expression data, and whether such an approach would yield fewer false positives than achieved by grouping genes into sets with clustering. Seriation is a statistical method for simultaneously ordering rows and columns of a symmetrical distance matrix for the purposes of revealing an underlying one-dimensional structure [13]. An assumption in seriation analysis is that there is an order (or distinct sub orders) in the data that are biologically meaningful. The inherent orders may represent any sequential structure among the data (e.g. their dependence on time or another variable). Seriation in its different flavors has been successfully applied in multiple fields, including archeology, psychology, and operational research; for instance, in archaeology it has been used to uncover the chronological order of archaeological deposits [14], [15]. The application of seriation for the analysis of high throughput biological data has been limited. One application in gene expression analysis is finding an optimal leaf ordering of a hierarchical clusterogram [16], [17]. In these studies global seriation was conducted after hierarchical clustering to aid in finding an optimal solution. In contrast, the present study examines whether the detection of local ordered structures in the data can be used in place of clustering for the identification of co-expressed genes.

Since finding the exact solution to seriation is known to be a nondeterministic polynomial time (NP)-hard problem [18], several heuristics have been developed to achieve an acceptable ordering solution [17]. We developed an original seriation heuristic we term ‘progressive construction of contigs’, which is based on step-wise reordering of the correlation matrix to produce chains or ‘contigs’ of related correlation values. We applied the seriation heuristic to analyses of both simulated and experimental SAGE data and showed that our approach can be used to effectively identify groups of co-expressed genes, and the relationships among these groups in a robust manner. We found that seriation performed better than a SAGE-specific clustering method on SAGE data containing spurious expression patterns that would arise due to measurement uncertainties and small number of experimental conditions compared to the large number of genes [3], [5]. Global patterns in the data revealed by seriation are easily detectable by eye from the reordered correlation matrix and can be interpreted biologically.

## Results

### Seriation using the progressive construction of contigs heuristic

Motivated by the opportunity to improve upon current methods for analyzing large scale expression datasets, we set out to explore the use of seriation as a substitute for clustering for identifying co-expression patterns in SAGE data. Seriation seeks the best enumeration order among objects based on their similarity according to a chosen criterion. Since the problem is NP-hard, we developed a novel heuristic specifically for the SAGE data analysis task. The ‘progressive construction of contigs’ heuristic attempts to put the most similar objects side by side without breaking already established chains of closely related elements we term ‘contigs’. Here we use pairwise correlations between expression vectors (normalized tag counts for a particular tag across all libraries) as the criterion for defining similarities between tags; however, in principle, other similarity criteria can be used for this task. The pairwise correlations between tag expression vectors x and y are calculated using the standard correlation coefficient function, 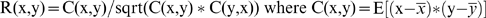 *x̅* and *y̅* are the means of expression vectors x and y, and E is the mathematical expectation. The correlation values are subsequently arrayed into a symmetric matrix, which is subjected to the following progressive seriation procedure.

In the first step, the tag pair with the highest correlation value is found and marked as the beginning of the first contig. At each subsequent step the tag pair with the next highest correlation value is identified. If one of the members of the tag pair is involved in a previously formed contig, the columns of the matrix are reorganized to place the other member at the nearest edge of the same contig; since the matrix is symmetrical, the rows are reordered accordingly. Importantly, previously reordered elements are kept intact in this process. If it is impossible to add the similarity maximum of the current step to a contig given the restriction on the previously-moved objects or if the tag pair with the correlation maximum does not involve any of the members of the formed contigs, the current similarity maximum is used to start a new contig. The seriation process continues until all elements have been processed. The result is the production of contigs of similar correlation values that can be displayed along the diagonal of the correlation matrix representing internal topologies in the data. Theoretically, in the case of a Robinson data structure, whereby the data are from a unimodal distribution, the contigs are merged into one and the obtained result is the most optimal single seriation solution [14], [17].

A key algorithmic difference between the seriation algorithm described above and a procedurally similar hierarchical clustering algorithm (such as the hierarchical clustering method developed in [19] and implemented in [4]) is the treatment of vectors after the highest pairwise correlation value has been identified at each step. In clustering, the vectors are averaged together into a new vector using a linkage rule (for instance, average linkage clustering) and this new vector is represented by a node in the hierarchical clusterogram. In contrast, in the case of seriation, no new vector or node is formed, and the rows and columns of the correlation matrix are merely reordered to reflect underlying patterns in the data as described above. Therefore, no linkage rule is required in seriation in addition to the distance metric used to define similarities.

In the current implementation of the seriation algorithm, ordered structures (contigs) are revealed by color-coding the reordered correlation matrix according to the magnitude of the correlation value. In this manner, visual inspection of the matrix allows for the selection of ordered contigs for further inspection. Due to the visualization component, the algorithm is able to analyze up to 4000 genes at a time (tested on 1.7 IBM PC Pentium 4, Z60t laptop) and is suitable for the analysis of pre-selected sets of genes. Importantly, the algorithm produces a robust solution for each seriation run (in other words, equivalent solution is produced upon repeated seriation of the same data set).

### Performance of seriation on simulated SAGE data

To test the performance of the seriation heuristic we generated a simulation dataset containing 500 expression vectors of dimension 5 (corresponding to 500 SAGE tags expressed over 5 different time points or conditions). Since expression data for a gene collected under different experimental conditions or at different time points are not completely independent, distinguishing genes with similar expression profiles in which the dynamics of gene expression changes is considered is of biological interest [5]. We designed the expression vectors to represent 10 different expression profiles that might be of potential biological interest (Figure S1).

**Figure 1:**
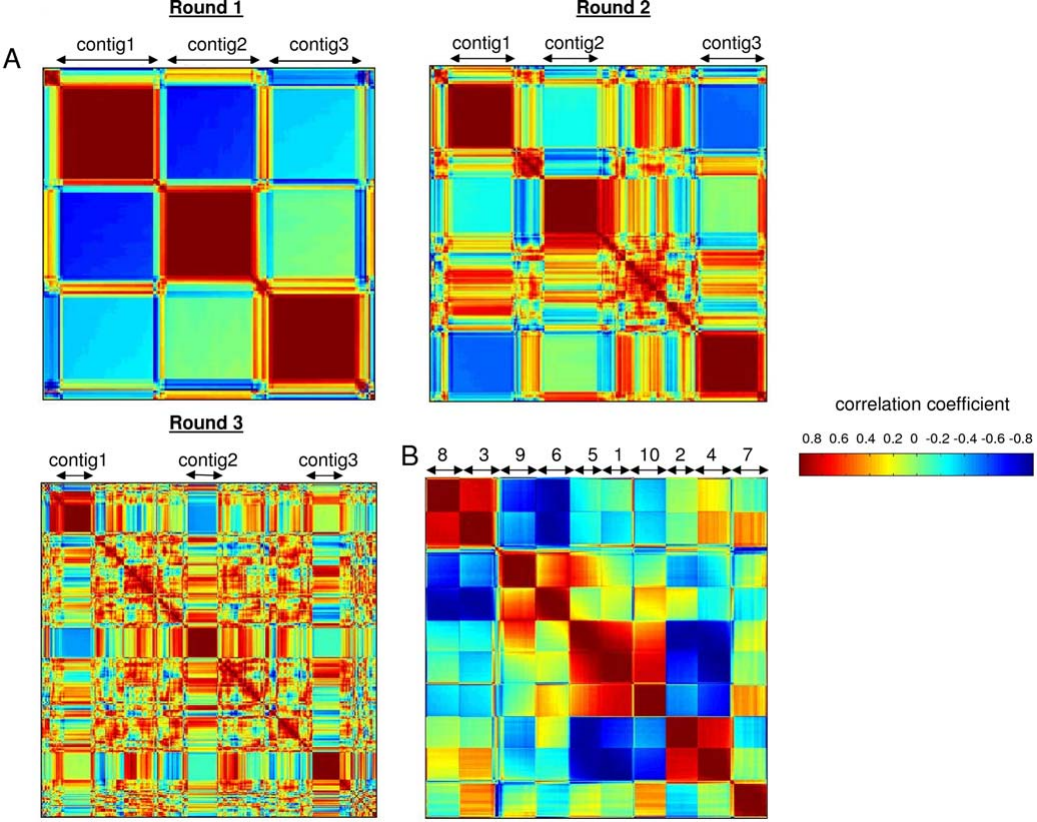
Performance of seriation on simulated SAGE data. (A). Seriation results of the three rounds of simulations with increasing amounts of noise from round 1 (34 tags) to round 3 (384 tags). The dark red squares along the diagonal indicate tags with the expression patterns 1–3 that were grouped together by seriation. (B). Seriation of 10 expression profiles with limited amount of noise. The dark red squares along the diagonal indicate tags in each expression profile that were grouped together. The numbers indicate expression patterns from Figure S1 that were grouped into each contig. Note that two contigs in the middle (5 and 1) appear more similar to each other than any other contig pair indicating similarity of the corresponding expression patterns.

To test the dependence of algorithm performance on the amount of noise in the data, we initially seriated three of these expression profiles with increasing numbers of noise tags. Pattern 2 corresponds to tags whose expression slightly peaks at time point 2 and then at time point 5; pattern 3 includes tags with a single expression peak at time point 2; and pattern 1 corresponds to tags with an expression peak over time points 3 and 4 (Figure S1). To closely simulate actual SAGE data, we added ‘noise’ or singleton tags whose expression profiles do not conform to any of the three patterns. Such expression profiles are common in gene expression datasets, particularly ones with few experimental conditions sampled relative to the number of genes [5]. Since it has been previously shown that SAGE data can be approximated by a Poisson distribution [12], we used Poisson-based rules for our simulations (see methods). Genes with similar expression profiles were modeled by a Poisson distribution with the same λ [12]. In contrast, genes that do not belong to any of the three patterns of interest (i.e. noise) were simulated by constructing expression profiles based on a Poisson distribution with random λ, obtained from a uniform distribution [1, 300]. We tested the performance of seriation as well as the PoissonC clustering algorithm, a successful K-means clustering algorithm previously developed specifically for SAGE data [12] on the simulation data set in three rounds, each time increasing the amount of noise present among the profiles of interest (Table S1). In each round, seriation yielded three clear contigs along the diagonal corresponding to the three patterns of interest (Figure 1A). Importantly, increasing the amount of tags corresponding to noise from 34 (round 1) to 384 (round 3) did not significantly affect the performance of the seriation algorithm (Table 1). We also applied the PoissonC algorithm to the simulation data using the same design. The optimal value of K was determined as described [20] and was set to K = 4 for each round. Values of K = 5 and K = 6 were also tested for round 2 and round 3 simulations, but did not produce significantly different results from those generated with K = 4. Interestingly, the performance of PoissonC declined with increasing amounts of noise (Table 1) illustrating the common problem with clustering analysis of gene expression data sets [3].

**Table 1:** Effect of the amount of noise in SAGE data on the performance of seriation and PoissonC.

		Pattern1		Pattern2		Pattern3	
		TP	FP	TP	FP	TP	FP
Round 1: 34 noise tags	Seriation	41	1	38	4	37	4
	PoissonC	41	2	38	2	37	5
Round 2: 120 noise tags	Seriation	41	6	38	6	37	3
	PoissonC	41	13*	38	14*	37	15*
Round 3: 384 noise tags	Seriation	41	5	38	3	37	3
	PoissonC	41	43*	38	99*	37	61*

Seriation and PoissonC were applied to a simulated SAGE data set containing three expression patterns and increasing amount of noise tags. The dataset is described in more detail in the text and in Table S1. TP (True Positives) include tags that were correctly classified as belonging to the correct expression group (expression pattern 1, 2, or 3 or noise) by assigning them to the cluster (PoissonC) or contig (seriation) containing other members of the expression group. FP (False Positives) include noise tags that have been erroneously assigned to a cluster or contig with tags that conform to the expression pattern 1, 2, or 3.

* The false positive rate is significantly higher for the PoissonC algorithm than it is for seriation mostly due to the erroneous assignment of noise tags to an expression pattern (p<0.05).

Overall, it can be noted that both algorithms performed well on data with relatively little noise (round 1); however, as the amount of noise in the data increased, seriation appeared more robust than clustering at identifying correct expression groupings. Importantly, both algorithms correctly grouped tags with similar expression profiles together in all three rounds (true positives), and the reduction in performance of PoissonC was due to an increase in false positives being incorporated into co-expression clusters.

Having established the excellent performance of seriation on noisy SAGE data containing a few expression profiles of interest, we went on to evaluate the dependence of performance on the number of expression profiles to be identified in the analysis. For this experiment, we used 10 expression profiles (Figure S1) and conducted the analysis as described above. We seriated 50 tags corresponding to each of the 10 expression profiles and 50 tags corresponding to noise. The resulting color-coded correlation matrix is shown in Figure 1B, and the comparative performance of PoissonC and seriation on the data is summarized in Table 2. The 10 expression profiles were grouped into 10 contigs along the diagonal by seriation. In addition to reordering tags according to the correct expression profile, seriation analysis was able to detect similarities among the profiles themselves. For instance, two squares in the middle of the matrix are distinct yet appear more similar to each other than any other pair of consecutive contigs. These contigs correspond to profiles 5 and 1 which are indeed very similar (Figure S1). Such additional information can not be revealed by clustering with PoissonC.

**Table 2:** Comparative performance of seriation and PoissonC on a simulated SAGE data set with 10 expression patterns.

Algorithm	TP	FP
Seriation	549	1
PoissonC	528	22*

Seriation and PoissonC were applied to the analysis of a simulated SAGE data set containing 10 expression patterns each including 50 tags, and 50 noise tags. TP (True Positives) are tags that were correctly classified as belonging to the right expression pattern or noise. FP (False Positives) are tags that were assigned to the wrong pattern or noise tags that were assigned to an expression pattern.

* The false positive rate is significantly higher for the PoissonC algorithm than it is for seriation (p<0.05).

### Performance of seriation on previously-published experimental SAGE data

**Figure 2:**
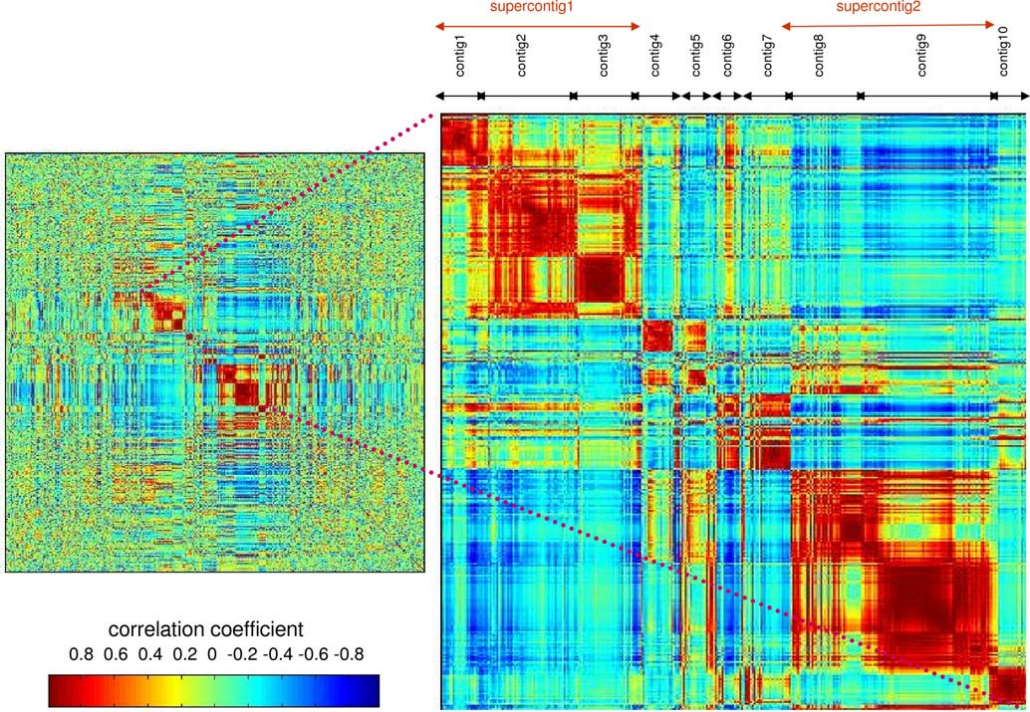
Seriation of genes expressed in mouse retinal SAGE libraries. SAGE data from Blackshaw et al. [20] were subjected to seriation analysis as described in the text. The resulting reordered correlation matrix containing correlation coefficients for each tag pair computed to measure the similarity of their retinal expression profiles is color-coded red to blue to represent decreasing correlation values. Ten contigs, including two supercontigs, recognizable as the squares of high (red) correlation values along the diagonal, are evident from the color-coded correlation matrix. The Figure on the right provides a zoomed-in view of the contigs.

**Figure 3:**
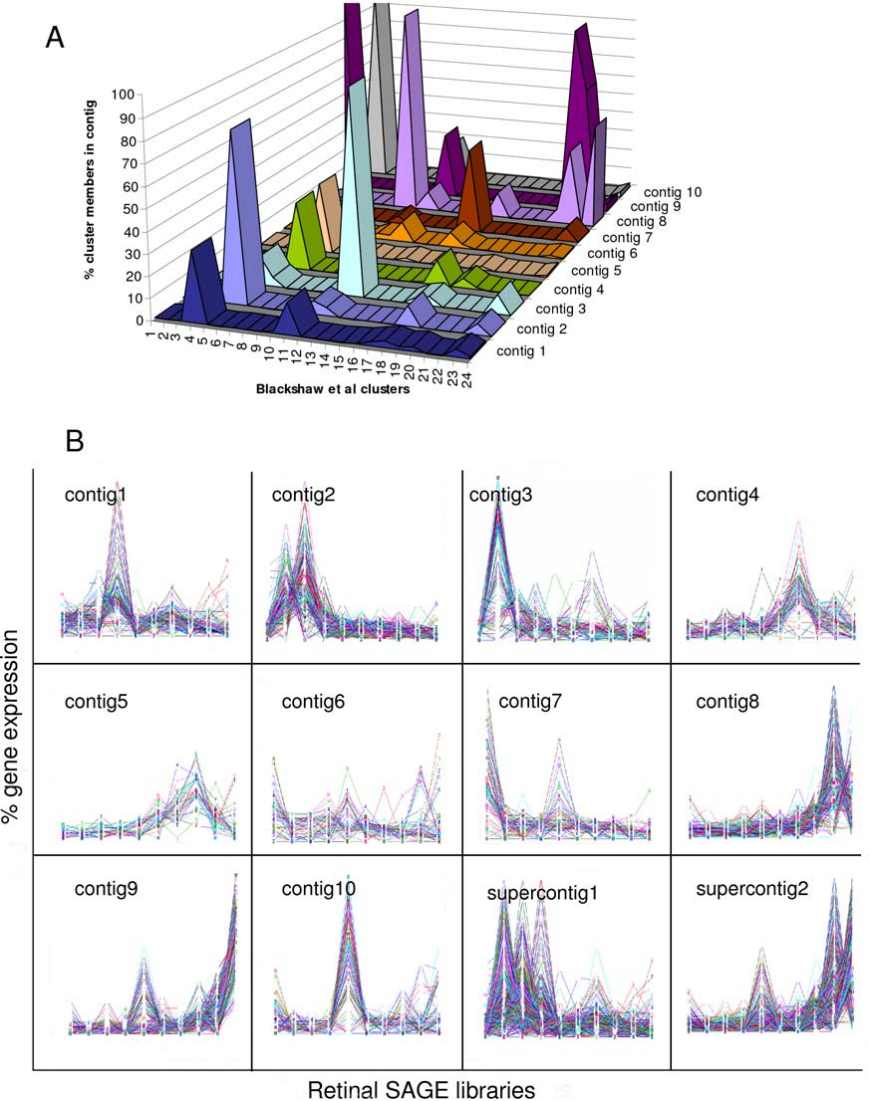
Analysis of seriation contigs of genes expressed in mouse retinal SAGE libraries. (A). Comparison of seriation contigs to the original clusters from Blackshaw et al. [20]. Seriation contigs are color-coded and plotted on the x-axis of the 3D graph. The peaks on the z-axis represent the percent cluster members (y-axis) present in the particular contig. Most seriation contigs are composed of one or several predominant clusters (also see Table 3). (B). Expression profiles of genes in seriation contigs. The relative expression levels from 0% to 100% are plotted on the y-axis for each contig while the retinal libraries derived from developmental stages E12.5, E14.5, E16.5, E18.5, P0.5, P2.5, P4.5, P6.5, P10, and adult are on the x-axis. The ordering of contigs is temporal such that genes expressed in earlier developmental stages tend to be in the first contigs, while genes expressed in later stages are in later contigs. This partitioning is particularly evident from the expression patterns of genes in the supercontigs.

To test the performance of seriation on previously analyzed experimental SAGE data, we applied the algorithm to reorder genes expressed in mouse retinal SAGE libraries based on similarity of their expression profiles [20]. The SAGE data were generated from mouse retinal tissues at 10 different developmental stages ranging from E12.5 (Theiler stage 20) to post natal day 10 (P10) and adult; the data were originally analyzed using the PoissonC algorithm with K = 24. We subjected the same dataset to seriation using the progressive construction of contigs procedure. The algorithm produced 10 contigs, including 2 contig groupings called ‘supercontigs’ (Figure 2). Most of the contigs were composed of members of one or several clusters from Blackshaw et al. [20] (Figure 3A; Table 3). The expression profiles of genes in the 24 clusters generated by Blackshaw et al. [20] are provided in Figure S2. It can be noted that as a result of seriation, clusters with similar expression patters were grouped together into contigs. For instance, clusters 8, 22, and 24, which contain genes whose expression peaks at post natal day 10, were grouped into contig8. Similarly, contig9 included clusters 1, 10, 22, and 24, which contain genes that are highly expressed in the adult library (Figure 3). For a full list of contig memberships of genes expressed in the retinal libraries see Figure S3.

**Table 3:** Comparison of seriation and PoissonC analyses of genes expressed in retinal SAGE libraries.

Contig	Predominant clusters*	Percent of predominant cluster members in contig	Percent of contig members in predominant cluster	Top Gene Ontology annotations enriched in predominant clusters*	Top Gene Ontology annotations enriched in contigs
Contig1	4	32.59%	58.4%	Mitochondrial	Ribonucleoprotein complex, p = 8.68E-03
Contig2	5	79.77%	74.19%	Ribosomal	Cytosolic ribosome (*sensu Eukarya*), p = 2.52E-09
Contig3	12	96.36%	42.74%	None	Biosynthesis, p = 1.11E-02
Supercontig 1 (contigs 1, 2, 3)	4, 5, 12	33.48%, 93.64%, 100%	17.24%, 37.24%, 12.64%	Mitochondrial, Ribosomal	Ribonucleoprotein complex, p = 7.17E-14
Contig4	6	31.94%	32.86%	RNA processing	Ligase activity, p = 3.28E-02
Contig5	6	33.33%	68.57%	RNA processing	N/A
Contig6	N/A	N/A	N/A	N/A	Structural molecule activity, p = 3.29E-05
Contig7	15	41.07%	67.65%	Ribosomal	Cytosolic ribosome (*sensu Eukarya*), p = 4.89E-07
Contig8	8, 22, 24	100%, 37.5%, 51.56%	15.09%, 2.8%, 46.7%	Ribosomal, Vision, Vision	Metal ion binding, p = 2.97E-03
Contig9	1, 10, 21, 22	100%, 32.2%, 92.86%, 62.5%	4.27%, 40.28%, 43.13%, 4.73%	Vision, Transporter activity, Vision, Vision	Vision, p = 4.74E-15
Supercontig 2 (contigs 8, 9)	1, 8, 10, 21, 22, 24	100%, 100%, 41.67%, 100%, 100%, 56.25%	2.13%, 7.57%, 26%, 23.17%, 3.78%, 25.53%	Vision, Ribosomal, Transporter activity, Vision, Vision	Perception of light, p = 7.77E-14
Contig10	2	100%	15%	Lens proteins	Structural constituent of eye lens, p = 1.17E-19

Column 2 contains Blackshaw et al. [20] clusters that are predominant in seriation contigs in column 1. GO categories enriched in clusters/contigs were determined using EASE software [34]; p<0.05.

* Data from Blackshaw et al. [20].

Significantly, Gene Ontology (GO) enrichment analysis indicated that clusters grouped into the same contig contained the same or similar enriched GO categories (Table 3) suggesting that they were somewhat functionally redundant. As an example, all but one of the clusters that fell into contig9 had ‘vision’ as the top enriched GO category. In addition to ordering co-expressed genes to form a contig, seriation provides insight into the relationship among the contigs. Here, it can be noted that the left-most contigs tend to contain genes whose expression peaks at earlier developmental stages, whereas the right-most contigs contain genes whose expression peaks in the late postnatal or adult lilbraries (Figure 3B). This ordering of contigs is temporal and biologically relevant, since the enriched GO categories of neighboring contigs are related. For instance, contigs 1, 2 and 3 that form supercontig1 are all enriched in genes that have a ribosomal function. Similarly, contigs 8 and 9 (supercontig2) are highly enriched in genes that function in vision (Table 3). Therefore, we argue that seriation provides an overview of the global biologically-relevant patterns in the data. Here, the results indicate that the retinal tissues contain two highly represented functional groups of genes, those involved in the ribosome functionality and those related to vision and light perception. These groupings are easily recognized from the color-coded seriated correlation matrix (Figure 2) as the two supercontigs. While it is possible to extract similar information from the clustering results, seriation provides a means to organize it in a relevant easily-interpretable and visualizable manner.

As evident from the simulation study, seriation is more discriminative than clustering analysis at grouping co-expressed genes together resulting in more accurate results. On the other hand, clustering analysis forces all the tags to belong to a cluster thereby resulting in more false positives. Here, many genes in the seriation experiment were not captured in the contigs (Figure 2) as they are presumably not sufficiently similar to any of the patterns present in the contigs. It can be noted that all the GO categories that were found to be enriched in Blackshaw et al. [20] clusters were also present in the contigs (Table 3) suggesting that Blackshaw et al. [20] clusters were somewhat redundant and may contain false positives.

### Performance of seriation on novel experimental SAGE data

We next applied the seriation algorithm to the analysis of SAGE libraries we generated as part of the Mouse Atlas Project (www.mouseatlas.org). The Mouse Atlas Project aims to produce a collection of SAGE libraries derived from various mouse tissues representing different developmental stages, ranging from embryonic stem cells to post-natal day 84 [21]; currently the resource contains over 200 different libraries. Due to our interest in the transcriptional regulation of pancreatic development we focused on analyzing the expression of transcription factors expressed in six SAGE libraries representing various stages of pancreatic endocrine cell development ranging from Theiler stage 17 (TS17) to post-natal day 70 (P70). Transcription factors are regulatory proteins that are presumed to be responsible for the coordinated expression of functionally-related genes. Transcription factors are at the top of the regulatory hierarchies that drive pancreatic development and enable beta cell maturation [22]. Thus, global analysis of transcription factor expression may provide insight into the mechanisms of pancreatic development and the misregulation of the mechanisms in disease.

**Figure 4:**
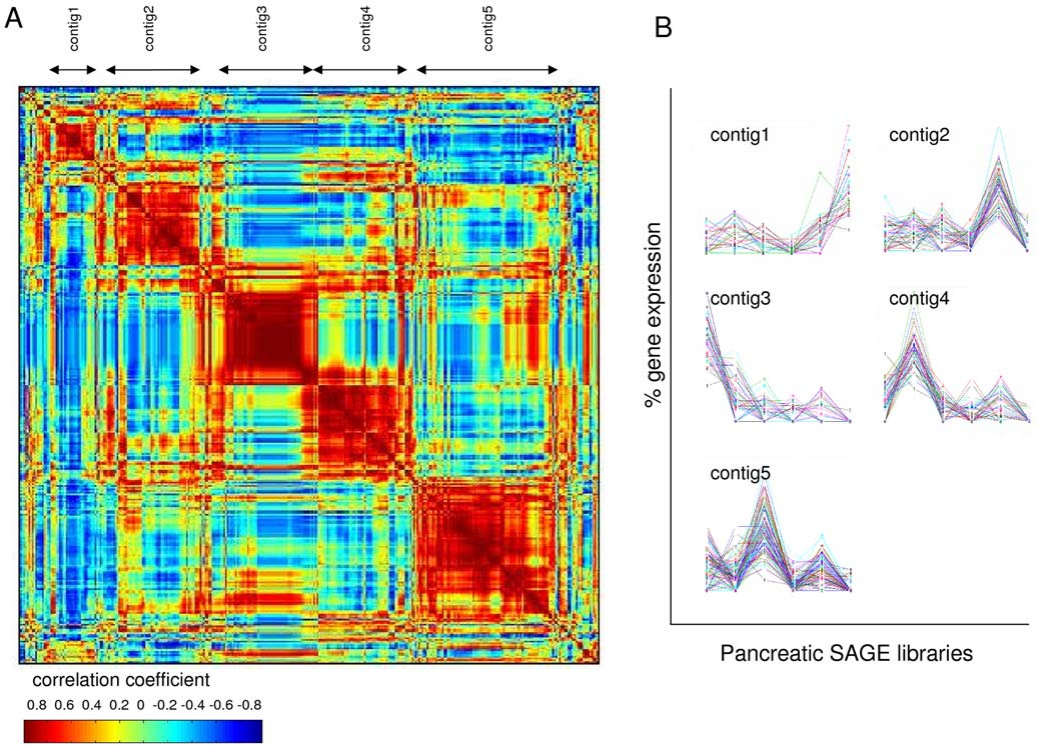
Seriation of transcription factors expressed in Mouse Atlas pancreatic libraries. SAGE data for transcription factors expressed in the pancreatic libraries from the Mouse Atlas project were subjected to seriation analysis as described in the text. The reordered correlation matrix containing correlation coefficients for each tag pair computed to measure the similarity of their pancreatic expression profiles is color-coded red to blue to represent decreasing correlation values. (A). 5 contigs recognizable as red squares along the diagonal are evident. (B). Expression profiles of transcription factors in contigs in (A). The relative expression levels from 0% to 100% are plotted on the y-axis for each contig while the pancreatic libraries derived from stages TS17, TS19, TS20, TS21, TS22, and P70 are on the x-axis.

SAGE expression profiles of 319 transcription factors expressed in six pancreatic libraries were subjected to seriation analysis. The algorithm yielded five contigs of transcription factor SAGE tags with similar expression profiles (Figure 4). For this analysis, we chose contigs as groupings of co-expressed genes (red squares along the diagonal, Figure 4A) with at least 10 members. Contigs of transcription factors expressed in the pancreatic libraries are provided in Figure S4. Annotation analyses of the resulting contigs suggested that they were functionally relevant based on the enrichment for GO category, SwissProt keyword and Kyoto Encyclopedia of Genes and Genomes (KEGG) annotations (Table 4, Figure S5). It was also evident that the contigs contained transcription factors that were expected to be grouped together based on their known membership in the same pathway. For instance, transcription factors implicated in islet cell type specification as part of FoxO signaling, Neurod1 and Foxa2 [23] were grouped together into contig1. In addition, Pax6 which was recently shown to be a target of Neurod1 [24] was placed in the same contig. Similarly, Neurogenin-Neurod cascade members implicated in endocrine development [25] together with a downstream transcription factor Nkx2-2 [26] were grouped into contig2. Smad3 and Smad4, known TGF-beta targets [23] were placed into contig4, which was enriched for TGF-beta signaling pathway annotation. These results suggest that transcription factor groupings produced by seriation are biologically relevant and recapitulate the transcriptional circuitry involved in the control of pancreatic development.

**Table 4:** Summary of seriation analysis of transcription factors expressed in pancreas.

Contig	Tags in contig	Characterized transcription factors in contig	Gene Ontology annotations enriched in contig*	SwissProt keywords enriched in contig*	KEGG annotations enriched in contig*
Contig1	28	**Foxa2, Neurod1**, **Pax6**, Myst1, Ets2, Mxd4, Mnt	Anatomical structure development, p = 1.52E-03; System development, p = 2.98E-03; Organ development, p = 5.91E-03	N/A	N/A
Contig2	55	Kin, Pole4, Foxa3, Tox3, **Neurod2**, **Neurog3**, Fos, Fev, Yy1, Jun, **Nkx2-2**	Defense response, p = 2.21E-02; Receptor activity, p = 2.13E-02	N/A	N/A
Contig3	61	**Hoxb7**, Dr1, Foxm1, Hmga1, Klf6, Snai1, **Hoxb5**, Sox18, **Hoxb1**, **Hoxb6**, **Hoxa10**, Hand1, **Pax1**, **Hoxa5**, **Hoxa7**	Multicellular organismal development, p = 1.45E-06; pattern specification process, p = 4.64E-05; regulation of cell differentiation, p = 9.97E-03	Zinc-finger, p = 1.46E-03; Homeobox, p = 2.19E-03	Adherens junction, p = 4.25E-02
Contig4	52	Hmga1, Foxp1, Mum1, Lin28, Msh6, Dnase2a, Mxd3, Rest, Gata4, Klf4, Cdx2, **Smad4**, **Smad3**	Transmembrane receptor protein serine/threonine kinase signaling pathway, p = 2.96E-02; regulation of signal transduction, p = 2.12E-02; negative regulation of cellular process, p = 2.14E-02	N/A	TGF-beta signaling pathway, p = 7.66E-04; Wnt signaling pathway, p = 3.65E-02
Contig5	97	**Pax4**, Dpm1, Sox9, Stat2, Arid2, Terf1, Dpm1, Elf3, Mtf1, Lass2, Arid3a, Stat4	Apoptosis, p = 1.23E-03; Programmed cell death, p = 1.43E-03; Regulation of apoptosis, p = 2.19E-03	Apoptosis, p = 6.08E-03; Coiled coil, p = 6.28E-03	N/A

Number of tags falling into each seriation contig is shown along with the representative contig members and their representative functional annotation using GO categories, SwissProt keywords, and KEGG pathways. For a full list of annotations enriched in the contigs see Figure S5. Known regulators of pancreatic development as well as the transcription factors discussed in the text are bolded.

* GO, SwissProt keyword, and KEGG pathway annotations enriched in contigs were computed using web-based FatiGO+ tool [35] and are provided with raw scores.

Previous studies have shown that genes with similar expression profiles are functionally related; moreover, co-expression has been reasonably successfully used to predict function of unknown genes [8], [27]. Therefore, the identified contigs of transcription factors can be used to gain insight into the functionality of unknown transcription factors in pancreatic development. For instance, Hand1, a major regulator of heart development which has been also implicated in vascular development [28], was placed into contig3 together with other basic helix-loop-helix (bHLH) transcription factors, Hox and Pax genes. Homeobox (Hox) and paired box (Pax) transcription factors have been presumed to function together to regulate a variety of developmental processes [29]. Our seriation analysis suggested that these transcription factors together with another bHLH family member Hand1 may function together during pancreatic development, a possibility that can be tested experimentally in the future. In addition, a crucial regulator of pancreatic development, Pax4, was placed into contig5 together with members of the Stat family of transcription factors, suggesting a potential interaction between Pax4 and the JAK/STAT pathway.

## Discussion

Clustering analysis has been the approach of choice for most gene expression studies. However, due to high dimensionality of gene expression datasets, many clustering algorithms are prone to producing false positive expression-based interactions [3]. SAGE data have been particularly poorly exploited by statistical analyses owing to the domination of the gene expression field by microarrays that produce continuous data as opposed to discreet count-derived data produced by SAGE. With the advent of next-generation sequencing technologies, sequence tag-based methods have been gaining popularity for gene expression analysis thereby necessitating the development of statistical methods for analyzing discreet expression data. To date, a few clustering algorithms designed to exploit the digital data structure have been developed for SAGE data analysis, and shown to perform favorably compared to conventional microarray clustering algorithms [12]. However, these methods are still subject to the inherent limitations of the clustering approach itself.

We explored the use of local seriation for the identification of co-expression patterns in SAGE data. The primary goal of seriation methods is finding an optimal ordering of a set of objects based on a similarity criterion. Since there are n! ways to order a set of n objects, finding the most optimal seriation order becomes computationally expensive with the increasing size of the data set; therefore, heuristics have been developed to achieve an optimal ordering solution [17]. We developed a novel bottom-up heuristic we termed ‘progressive construction of contigs’ specifically designed for seriation of gene expression vectors according to their similarity. The ‘progressive construction of contigs’ heuristic is based on a greedy process that does not question the previous steps, and thus is fast and can, in principle, be implemented with large datasets. We tested the performance of seriation on both simulated and experimental SAGE data, and compared its performance with that of the PoissonC K-means clustering algorithm, a current state-of-the-art method in the field of SAGE data analysis [12]. We demonstrated that seriation was able to identify contigs of co-expressed genes that were related to clusters of co-expressed genes obtained by PoissonC (Table 3). We showed that the co-expression contigs were enriched for genes with similar functions as defined by both Gene Ontology and SwissProt keyword annotations as well as the known memberships in the same pathway. Therefore, we provided an empirical demonstration that the results from the two approaches are related and are complementary to each other. We further showed that in contrast to clustering, seriation could detect relationships among contigs of co-expressed genes, such as their temporal order, whenever such relationships were present in the data. Moreover, based on the simulation results, seriation appeared less sensitive to noisy data than PoissonC, and produced fewer false positives.

The major conceptual difference between seriation and clustering underlying the differential performance of the methods on noisy SAGE data stems from the different primary goals of the two methods. The primary goal of seriation is reordering during which inherent patterns in the dataset (e.g., presence of groups of elements that are related to one another) are revealed. On the other hand, the primary goal of clustering is partitioning the dataset into groups of similar elements. A key advantage of ordering over grouping is that ordering allows for the discovery of gradual progressions in the data while such gradual information is lost in grouping analyses. For instance, Robinson properties in the data can be revealed by seriation but not by clustering [14]. Gene expression changes over various experimental conditions are often of a gradual nature rendering seriation a useful tool for the discovery of similar expression profiles. In other words, the identification of groups of related elements is a consequence of seriation while it is the primary goal of clustering. Due to this fact, following clustering analysis of gene expression datasets, all genes are assigned to the most appropriate cluster based on a generic linkage rule. In contrast, following seriation analysis that does not require a linkage rule, contigs of genes with high pairwise correlation coefficients are revealed by reordering. Real versus spurious co-expression interactions can be thus gauged from the color-coded reordered correlation matrix (e.g., Figure 1, Figure 2, Figure 4A) wherein clear tightly-formed red squares along the diagonal reveal groupings of co-expressed genes while the rest of the matrix represents tags that do not belong to any of the contigs.

Overall, we showed that seriation is a useful tool for pattern discovery and visualization in SAGE datasets. The method allows one to estimate the number of co-expression patterns present in the dataset (estimated from the number of formed contigs) as well as the amount of ‘noise’ or spurious expression profiles (estimated from the number of tags that do not appear to belong to a contig). We showed that seriation correctly detected groups of simulated expression profiles, correctly identified enriched GO categories obtained by PoissonC, and correctly revealed a number of known transcription factor interactions from pancreas SAGE data. Therefore, we suggest that the application of seriation for the identification of co-expressed genes in tag-based gene expression studies should be explored further.

## Materials and Methods

### Simulation study

The simulation design was influenced by a previously described design for short-term time series microarray expression data [5]. However, we used the Poisson distribution that has been shown to be suitable for modeling SAGE data [12] instead of the uniform distribution used in [5] to model microarray data. In brief, we generated a simulation dataset containing 500 expression vectors of dimension 5. The expression profiles for each tag (v_0_, v_1_, v_2_, v_3_, v_4_) belonging to one of 10 expression patterns defined by setting (m_0_, m_1_, m_2_, m_3_, m_4_) to (0, 1, 0, 0, 2) for pattern 1; (0, -1, 1, 1, -1) for pattern 2; (0, 2, 1, 0, 0) for pattern 3; (0, 1, -1, -1, 1) for pattern 4; (0, -1, 0, 0, -2) for pattern 5; (0, -2, -1, 0, 0) for pattern 6; (0, 1, -1, 1, -1) for pattern 7; (-1, 1, 1, -1, 0) for pattern 8; (2, 0, 0, 1, 0) for pattern 9; and (0, 0, 1, 2, 0) for pattern10 in the equation below, were determined as follows:
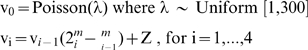
where Z takes on the values of -1, 0 or 1 with equal probability of 1/3 and represents errors in tag count measurements. λ was kept the same for all genes in the same expression pattern group as suggested in [12]. The resulting expression profiles of the 10 groups are displayed in Figure S1. The noise was modeled using the same rule as above but selecting a random λ ∼ Uniform [1, 300] to model the expression profile of each tag. Simulations were conducted over three rounds with increasing amount of noise (Table S1). Seriation algorithm was run several times for each round and produced the same result. PoissonC algorithm was run over 100 iterations and the iteration results were combined into consensus clusters.

### Experimental SAGE data

The retinal dataset was obtained from Blackshaw et al. [20] and was processed as described by the authors. The mouse pancreatic SAGE libraries SM161/SM244, SM231, SM243/SM160, SM225/SM249, SM232 and SM017 were obtained from the Mouse Atlas web site (www.mouseatlas.org). The libraries were built as described in [21] and in [30]. All tag processing, including the removal of linker-derived tags, quality filtering (95% sequence quality cutoff was used) and mapping was done in DiscoverySpace 4.0 software as described [31]. The tag counts in each library were normalized to the depth of 100,000.

### Mouse transcription factors

We obtained the list of mouse transcription factors by selecting Ensembl genes containing DNA-binding domains from Pfam [32], [33]. The sequences were selected based on the mouse genome NCBI build 32 and are analyzed in O. Morozova and T.R. Hughes. Patterns of transcription factor evolution in vertebrates. Proceedings of the Third Canadian Student Conference on Biomedical Computing (CSCBC), 2008. We found that out of 994 transcription factors expressed in the Mouse Atlas libraries, 319 were present in the pancreatic libraries with a tag count of 4 or higher.

### GO category, SwissProt keyword and KEGG pathway enrichment analysis

GO category analysis of retinal SAGE clusters and contigs was performed using EASE software as described [34]. GO category, SwissProt keyword and KEGG pathway enrichment analysis of transcription factor contigs was performed using the web-based FatiGO+ tool [35]. P-values of less than 0.05 were considered statistically significant for both analyses.

### Clustering analysis

K-means clustering analysis was performed according to the PoissonC algorithm [12]. The within-cluster dispersion was calculated as described [20]. The java implementation of the clustering algorithm and the within-cluster dispersion calculation was kindly provided by Li Cai (Rutgers University, NJ).

### Seriation algorithm and its implementation

Seriation was conducted on simulated, retinal or Mouse Atlas SAGE data using the custom MATLAB implementation. The algorithm was run three times on each experimental dataset to ensure the seriation result was robust. The analysis of simulated SAGE data was done as described above. The implementation of the algorithm can be made available to interested academic users upon request.

## Supporting Information

Figure S1 Composition of the simulation dataset during three rounds of simulations.
(0.86 MB TIF)

Figure S2 Expression profiles of genes in 24 clusters from Blackshaw et al. [20]. The relative expression levels from 0% to 100% are plotted on the y-axis for each cluster while the retinal libraries derived from developmental stages E12.5, E14.5, E16.5, E18.5, P0.5, P2.5, P4.5, P6.5, P10, and adult are on the x-axis.
(2.79 MB TIF)

Figure S3 Contig membership of genes expressed in retinal SAGE libraries.
(0.12 MB XLS)

Figure S4 Contig membership of transcription factors expressed in pancreas.
(0.04 MB XLS)

Figure S5 Annotations enriched in contigs of transcription factors expressed in pancreas.
(0.03 MB XLS)

Table S1 Composition of the simulation dataset during three rounds of simulations. Simulated SAGE datasets were constructed to include three different expression patterns of potential biological interest (depicted in Figure S1, patterns 1, 2, and 3) and modeled as described in Materials and Methods. To simulate actual SAGE data, we included singleton tags that do not strictly conform to any of the three expression patterns (referred to as ‘noise’). The simulation was conducted over three rounds with constant numbers of tags in each expression category (rows 1–3) and increasing numbers of noise tags (row 4). The expression profiles in each category are shown in column 5 and explained in Materials and Methods.
(0.03 MB DOC)
