# Supplementary figures and images for: A Seriation Approach for Visualization-Driven Discovery of Co-Expression Patterns in Serial Analysis of Gene Expression (SAGE) Data
Source: PLoS One. 2008 Sep 12;3(9):e3205. doi: 10.1371/journal.pone.0003205 (PMC2527533; doi:10.1371/journal.pone.0003205)

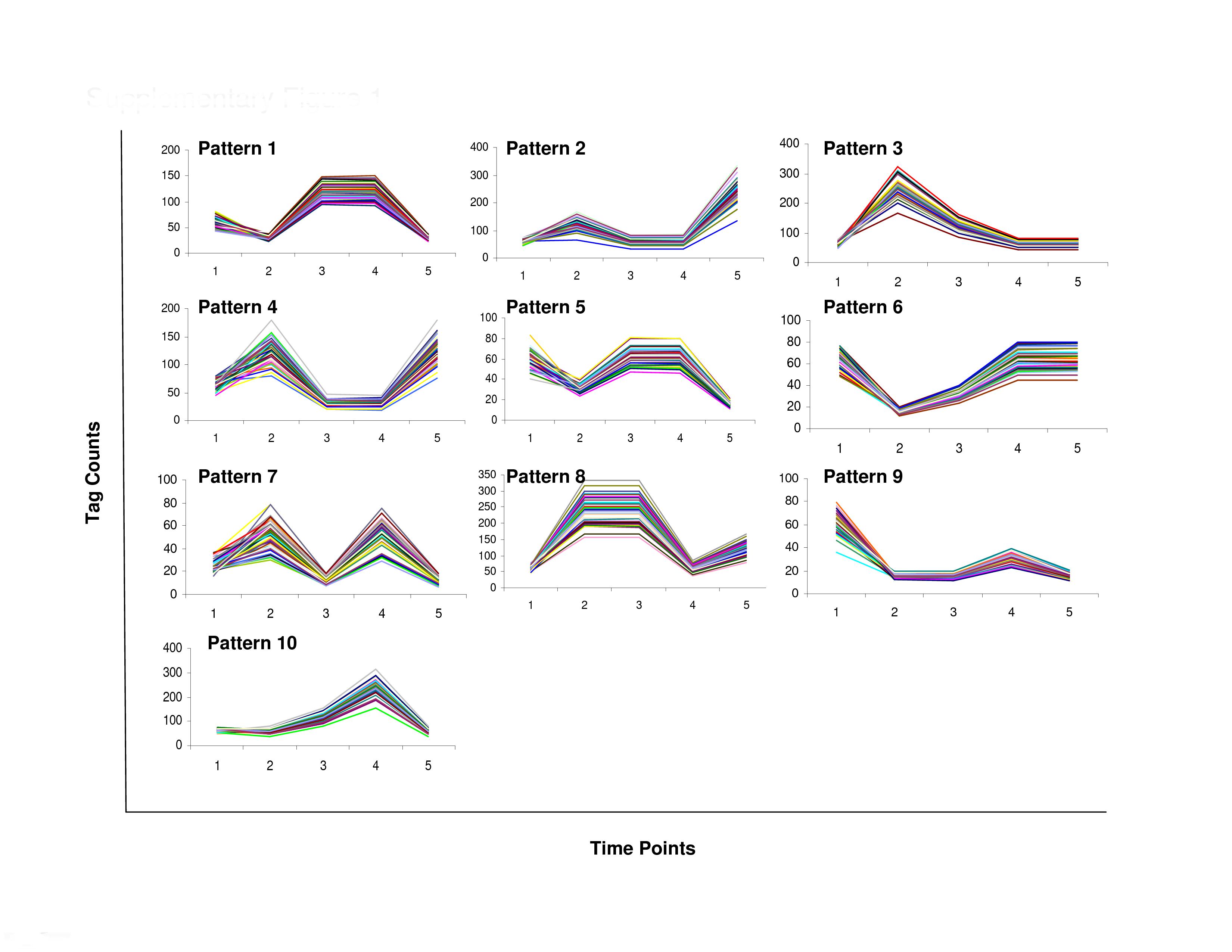

Supplement: Figure S1 — Composition of the simulation dataset during three rounds of simulations. (0.86 MB TIF) [file pone.0003205.s001.tif]

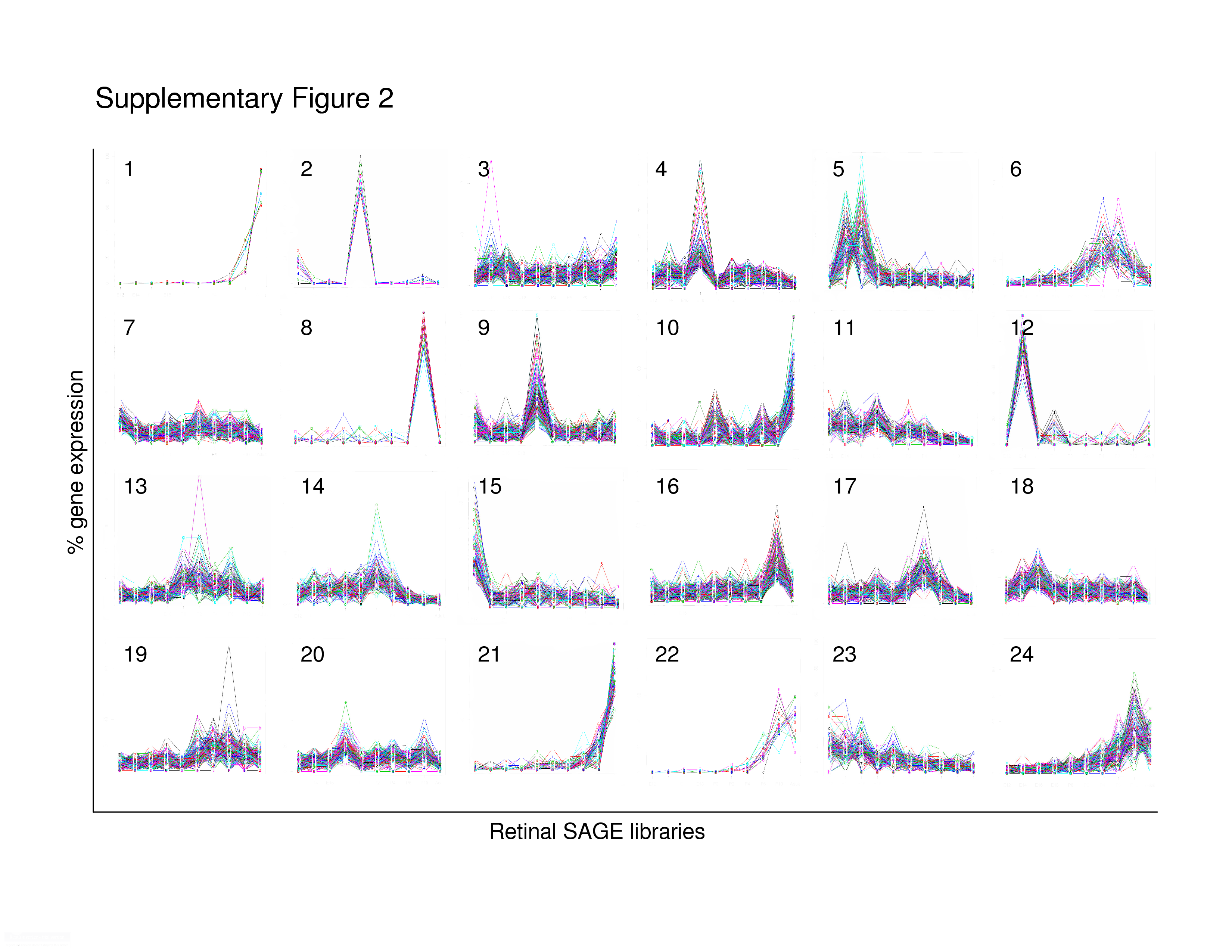

Supplement: Figure S2 — Expression profiles of genes in 24 clusters from Blackshaw et al. [20]. The relative expression levels from 0% to 100% are plotted on the y-axis for each cluster while the retinal libraries derived from developmental stages E12.5, E14.5, E16.5, E18.5, P0.5, P2.5, P4.5, P6.5, P10, and adult are on the x-axis. (2.79 MB TIF) [file pone.0003205.s002.tif]
