# Supplementary material for: A Seriation Approach for Visualization-Driven Discovery of Co-Expression Patterns in Serial Analysis of Gene Expression (SAGE) Data
Source: PLoS One. 2008 Sep 12;3(9):e3205. doi: 10.1371/journal.pone.0003205 (PMC2527533; doi:10.1371/journal.pone.0003205)
Supplement: Table S1 — Composition of the simulation dataset during three rounds of simulations. Simulated SAGE datasets were constructed to include three different expression patterns of potential biological interest (depicted in Figure S1, patterns 1, 2, and 3) and modeled as described in Materials and Methods. To simulate actual SAGE data, we included singleton tags that do not strictly conform to any of the three expression patterns (referred to as ‘noise’). The simulation was conducted over three rounds with constant numbers of tags in each expression category (rows 1–3) and increasing numbers of noise tags (row 4). The expression profiles in each category are shown in column 5 and explained in Materials and Methods. (0.03 MB DOC) [file pone.0003205.s006.doc]

**Table S1.** Composition of the simulation dataset during three rounds of simulations.

| Patterns | Number of tags (Round 1) | Number of tags (Round 2) | Number of tags (Round 3) |
| --- | --- | --- | --- |
| 1 | 41 | 41 | 41 |
| 2 | 38 | 38 | 38 |
| 3 | 37 | 37 | 37 |
| Noise | 34 | 120 | 384 |
| Total | 150 | 236 | 500 |

Simulated SAGE datasets were constructed to include three different expression patterns of potential biological interest (depicted in Figure S1, patterns 1, 2, and 3) and modeled as described in Materials and Methods. To simulate actual SAGE data, we included singleton tags that do not strictly conform to any of the three expression patterns (referred to as ‘noise’). The simulation was conducted over three rounds with constant numbers of tags in each expression category (rows 1-3) and increasing numbers of noise tags (row 4). The expression profiles in each category are shown in column 5 and explained in Materials and Methods.
